# Supplementary material for: Direct Visualization of Horizontal Gene Transfer by Transformation in Live Pneumococcal Cells Using Microfluidics
Source: Genes (Basel). 2020 Jun 20;11(6):675. doi: 10.3390/genes11060675 (PMC7350252; doi:10.3390/genes11060675)
Supplement: Supplementary file 1 [file genes-11-00675-s001.zip › Supplementary Materials.pdf]

Direct visualization of horizontal gene transfer by transformation in live pneumococcal cells using microfluidics

Isabelle Mortier-Barrière, Patrice Polard and Nathalie Campo

Supplementary Materials

Figure S1. Catalase and high flow rates promote pneumococcal growth in microfluidic chambers

Figure S2. Optimal competence induction in microfluidic chambers requires high concentrations of synthetic CSP

Figure S3. Kinetics of expression of P_E-*gfp* (strain R4254), P_E-*mTurquoise* (strain R4255) and P_E-*mCherry* (strain R4256)

Figure S4. Whole field of view of a representative image extracted at 133 minutes from time-lapse microscopy experiment shown in Figure 5

Table S1. Strains, plasmids, and primers used in this study

Video S1. Time-lapse microscopy of a mixed culture containing equal densities of strains R4254 (P_E-*gfp*), R4255 (P_E-*mTurquoise*) and R4256 (P_E-*mCherry*). Images were captured at 2 min intervals during 1 hour. The starting point of the time-lapse corresponds to the beginning of CSP injection. Time is shown in minutes.

Video S2. Time-lapses microscopy of a mixed culture containing equal densities of the two strains, R4254 (P_E-*gfp*) and R3728 (*dprA-gfp*). Images were captured at 50 sec intervals during 20 minutes. The starting point of the time-lapse corresponds to the beginning of CSP injection. Time is shown in minutes.

Video S3. Time-lapse microscopy of a mixed culture containing 5/6 of strain R3708 (*ftsZ*-stop-*gfp*) and 1/6 of strain R4256 (P_E-*mCherry*). Images were captured at 4-min intervals during 2.5 hours. The starting point of the time-lapse corresponds to the beginning of CSP injection. Time is shown in minutes.

Video S4. Time-lapse microscopy of a mixed culture containing 5/6 of strain R3708 (*ftsZ*-stop-*gfp*) and 1/6 of strain R4256 (P_E-*mCherry*). Images were captured at 4-min intervals during 2.5 hours. The starting point of the time-lapse corresponds to the beginning of CSP injection. Time is shown in minutes.

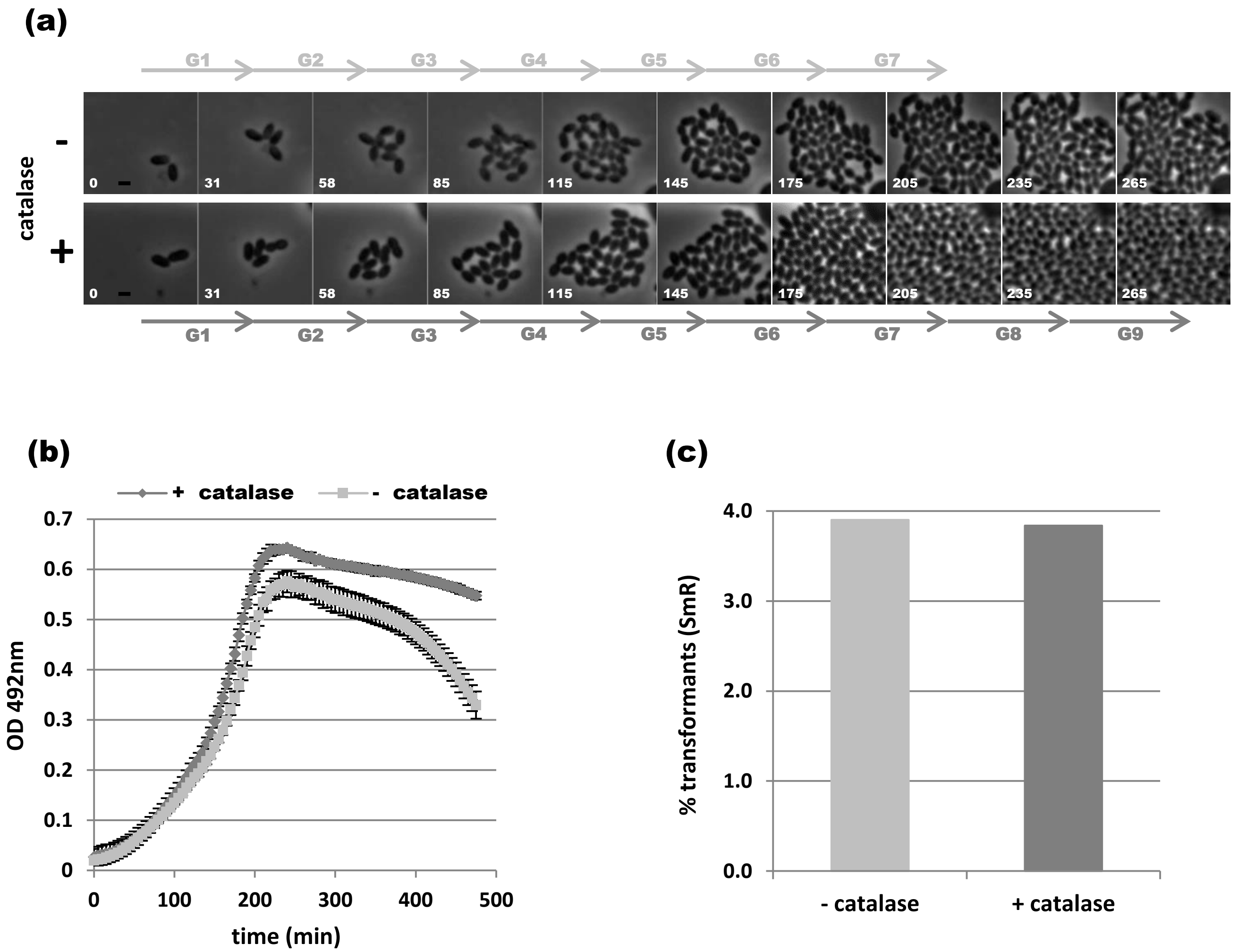

Figure S1. Catalase and high flow rates promote pneumococcal growth in microfluidic chambers. (**a**) Still images from time-lapse microscopy of R1501 cells grown in C+Y medium at 37°C under a flow rates of 6 µL/h (2psi) in the presence (bottom panel) or absence (top panel) of catalase (300 U/ml). Images were captured at 5 min intervals during 5 hours. Representative phase contrast images are shown. Time is indicated in minutes. G1 to G9 and arrows indicate successive cell division events. Note that cell growth stops after 7 cell division events without catalase (top panel). Scale bar, 1 µm; (**b**) Growth curves of strain R1501 at 37°C in C+Y medium in the presence (dark grey) or absence (light grey) of catalase. Averages of three replicates are shown. Error bars show standard deviations; (**c**) Comparison of chromosomal transformation frequency of strain R1501 grown at 37°C in C+Y medium in the presence or absence of catalase.

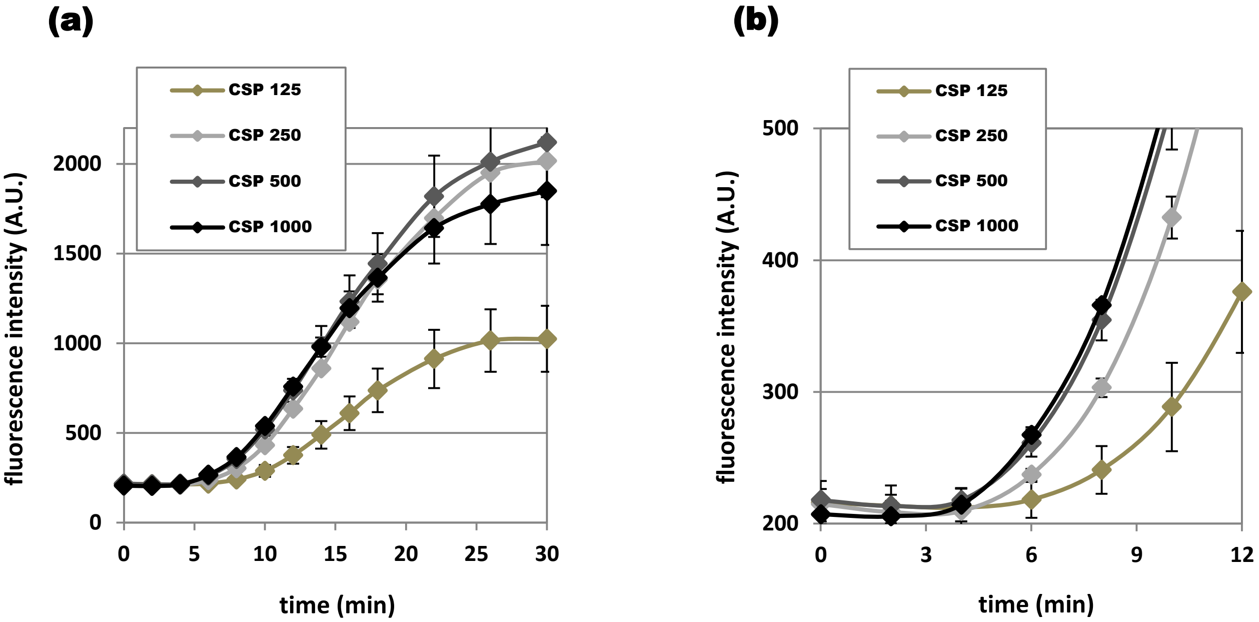

Figure S2. Optimal competence induction in microfluidic chambers requires high concentrations of synthetic CSP. (**a**) Kinetics of expression of P_E-*gfp* (strain R4254) in response to CSP addition at differing concentrations (125 ng/µL, 250 ng/µL, 500 ng/µL, 1000 ng/µL). Average fluorescence intensities per µm^2 are based on microscopy images and shown in arbitrary units (A.U.). More than 200 cells were analyzed per time point and per experiment. Time zero corresponds to the onset of CSP injection. Averages of three replicates are shown. Error bars show standard deviations; (**b**) As in panel (a) but with different x- and y-axis scales.

(a)

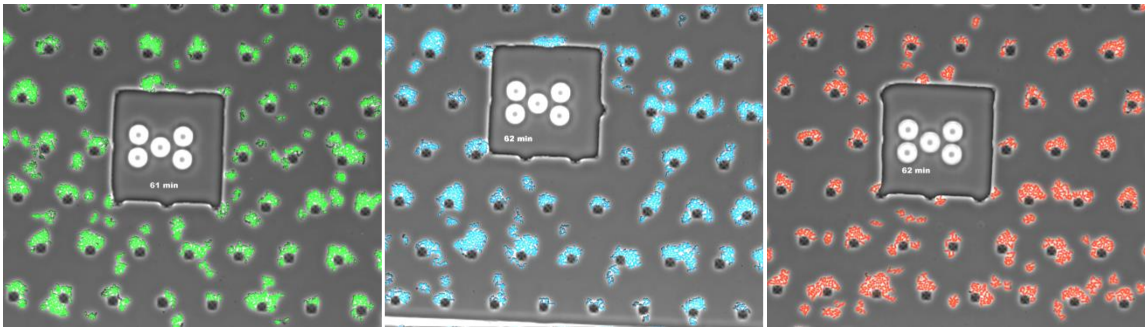

(b)

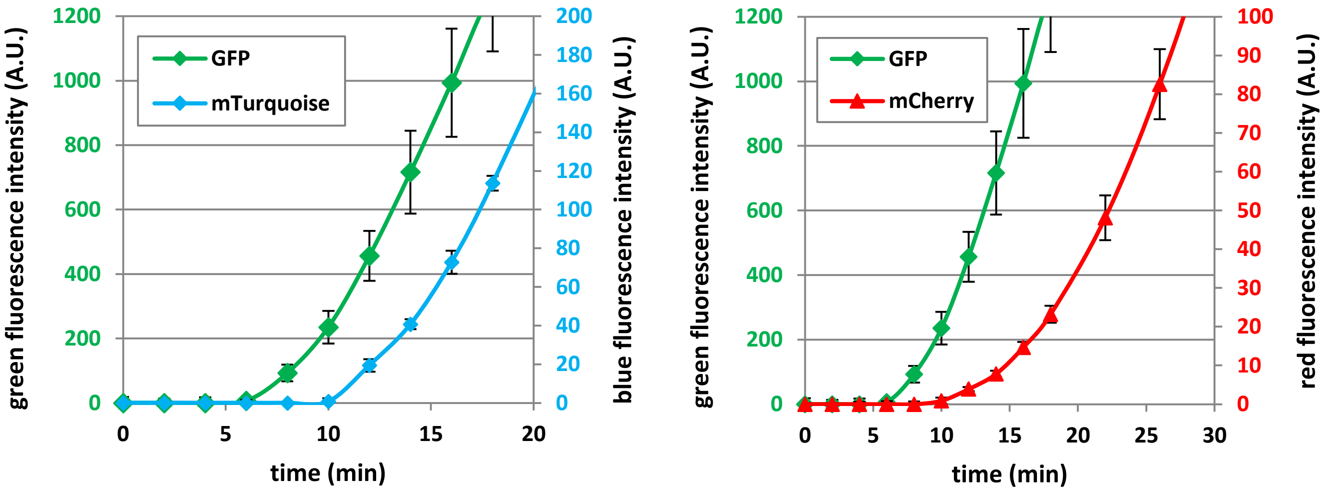

(c)

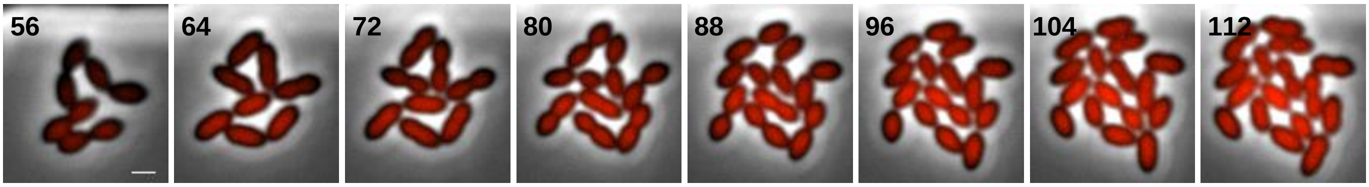

Figure S3. Kinetics of expression of P_E-*gfp* (strain R4254), P_E-*mTurquoise* (strain R4255) and P_E-*mCherry* (strain R4256). (**a**) Whole field of view of fluorescent images acquired 62 minutes after CSP injection. Overlays between phase contrast (grey) and GFP (green, *left*), mCherry (red, *middle*) and mTurquoise (blue, *right*) are shown; (**b**) Comparison of the kinetics of expression of P_E-*gfp* (strain R4254) and P_E-*mTurquoise* (strain R4255) (*left*); and P_E-*gfp* (strain R4254) and P_E-*mCherry* (strain R4256) (*right*); (**c**) Still images from time-lapse microscopy of R4256 cells (P_E-*mCherry*). Images were captured at 8 min intervals during 2 hours. The starting point of the time-lapse corresponds to the beginning of CSP perfusion. First image shown corresponds to the 56th minute. Time is indicated in minutes. Scale bar, 1 µm

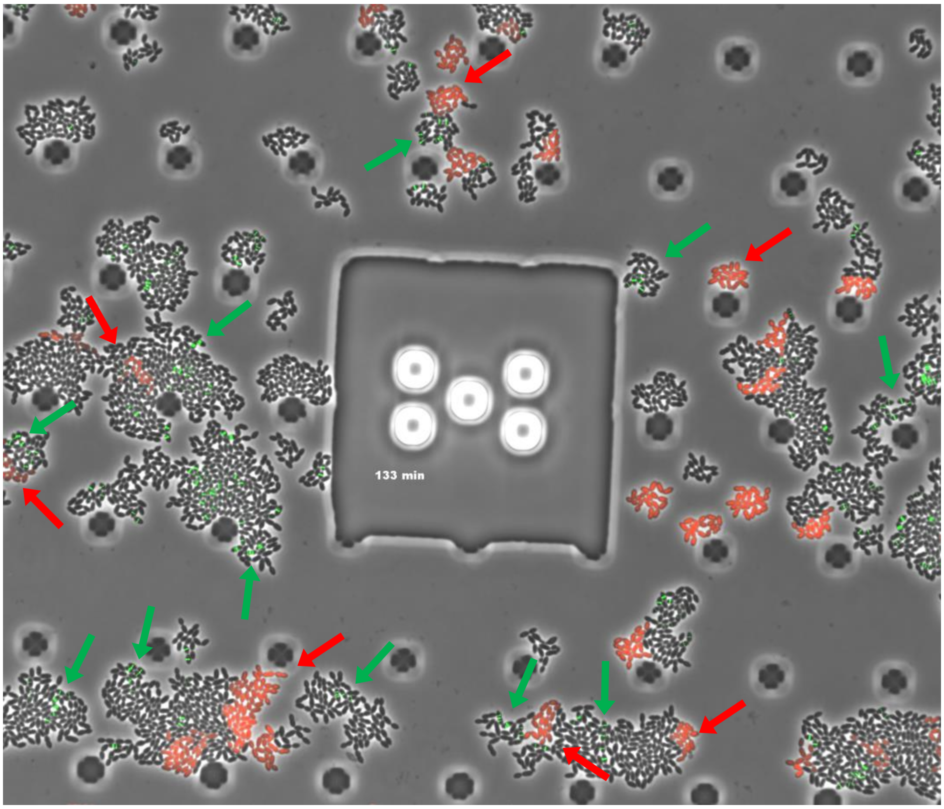

Figure S4. Whole field of view of a representative image extracted at 133 minutes from time-lapse microscopy experiment shown in Figure 5. Overlay between phase contrast (grey), GFP (green) and mCherry (red) is shown. Red arrows indicate cells expressing mCherry (strain R4256, P_E-*mCherry*) and green arrows indicate cells expressing FtsZ-GFP (strain R3708 transformed with DNA carrying a functional *ftsZ-gfp* fusion).

Table S1. Strains, plasmids, and primers used in this study.

Strains	Genotype/relevant features[a]	Source/Reference
R800	*wild-type* (R6 derivative)	[16]
R304	*nov1*, *rif23*, *str41*; Nov^R, Rif^R, Sm^R	[39]
R1501	Δ*comC*	[7]
R1502	Δ*comC*, *ssbB::luc* (*ssbB*$^+$, *cat*); Cat^R	[7]
R3702	Δ*comC*, *hexA*Δ3::*ermAM*, *ftsZ-gfp;* Ery^R	[12]
R3708	Δ*comC*, *hexA*Δ3::*ermAM*, *ftsZ-stop-gfp;* Ery^R	[12]
R3728	Δ*comC*, *dprA-gfp*	Johnston and Polard, to be published elsewhere
R4254	Δ*comC*, *ssbB::luc* (*ssbB*$^+$, *cat*); CEP_E-*gfp*(*Sp*) (*kan*), (from plasmid pIM122); Cat^R, Kan^R	This study
R4255	Δ*comC*, CEP_E-*mTurquoise*(*Sp*) (*kan*) (from plasmid pIM123); Kan^R	This study
R4256	Δ*comC*, CEP_E-*mCherry*(*Sp*) (*kan*) (from plasmid pIM124); Kan^R	This study

Plasmids	Description	Reference
pUC57-*gfp* (*Sp*)	pUC57 derivative carrying a 728-bp *Nco*I-*Bam*HI synthetic fragment containing the *gfp*(*Sp*) gene encoding GFP with codon optimized for *S. pneumoniae* R6; Ap^R	[19]
pUC57-*mTurquoise* (*Sp*)	pUC57 derivative carrying a 720-bp synthetic fragment containing the *mTurquoise*(*Sp*) gene encoding mTurquoise with codon optimized for *S. pneumoniae* R6; Ap^R	[17]
pUC57-*mCherry* (*Sp*)	pUC57 derivative carrying a 684-bp synthetic fragment containing the *mCherry*(*Sp*) gene encoding mCherry with codon optimized for *S. pneumoniae* R6; Ap^R	GenScript, USA
pCEP$_E$	pCEP derivative containing the ComE-dependent promoter, P_E, of the *comCDE* operon and the RBS of *comC*; Kan^R, Spc^R	[20]
pIM122	pCEPE derivative carrying the P_E-*gfp* transcriptional fusion, a 720 bp *Nco*I-*Bam*HI synthetic fragment containing the *gfp*(*Sp*) gene from pUC57-*gfp*(*Sp*)*;* Kan^R, Spc^R	This study

pIM123	pCEPE derivative carrying the P_E-*mTurquoise* transcriptional fusion, a 720 bp *Nco*I-*Bam*HI synthetic fragment containing the *mTurquoise*(*Sp*) gene from pUC57- *mTurquoise* (*Sp*); Kan^R, Spc^R	This study
pIM124	$pCEP_E$ derivative carrying the P_E-*mCherry* transcriptional fusion, a 684 bp *Nco*I-*Bam*HI synthetic fragment containing the *mCherry*(*Sp*) gene from pUC57-*mCherry*(*Sp*); Kan^R, Spc^R	This study

**Primers**	**Sequence**
oMB2	GAATTCCCATGGTTTCTAAAGGTG
oMB94	GCGCTGCAGGGGTGCAGGAGGTCAACCTGAGGTTGGTCGT
oMB97	CCAGGGATCCCGAACATCTATAATGACCTTATCCGTT
oCN87	CCAGGATCCTTATTTATACAATTCATCCATACC
oIM78	ATACCATGGTTTCTAAAGGTGAAG
oIM79	ATAGGATCCTTATTTATACAATTCATC
oIM80	ATACCATGGCAATCATCAAGGAATTTATGCG
oIM81	ATAGGATCCTTATTTGTAAAGTTCATCC

[a] ^{R}, resistance; Ap, ampicillin; Cat, chloramphenicol; Ery, erythromycin; Kan, kanamycin; Nov, novobiocin; Rif, rifampicin; Sm, streptomycin;
